# Parent-perceived barriers to accessing services for their child’s mental health problems

**DOI:** 10.1186/s13034-021-00357-7

**Published:** 2021-01-29

**Authors:** Anna Sofie Hansen, Gry Kjaersdam Telléus, Christina Mohr-Jensen, Marlene Briciet Lauritsen

**Affiliations:** 1 Psychiatry, Aalborg University Hospital, Aalborg, Denmark; 2 Department of Clinical Medicine, Aalborg University, Aalborg, Denmark; 3 Institute of Communication and Psychology, Psychology, Aalborg University, Aalborg, Denmark

**Keywords:** Child, Adolescent, Mental health, Service use, Help-seeking, Barriers

## Abstract

**Background:**

Many children and adolescents with impairing mental health disorders are not in contact with specialized child and adolescent mental health services (CAMHS). In order to close the service gap, it is important to increase our knowledge of barriers to access. The aim of this study was to investigate parent perceived barriers to accessing services for their child’s mental health problems to identify potential areas for improvement of access to CAMHS.

**Method:**

In this cross-sectional observational study 244 parents of children and adolescents referred to outpatient psychiatric assessment were interviewed using the Children’s services interview regarding barriers to accessing child mental health services across healthcare, educational services and social services. Parent reported barriers were analyzed in relation to the child’s age, referral reason, symptom duration and impairment of the child.

**Results:**

The most commonly reported barriers were lack of information about were to seek help (60.3%), the perception that professionals did not listen (59.8%) and professionals refusing to initiate interventions or provide referral to services (53.7%). Lack of knowledge, stigmatization and unavailability of services were common themes across barriers to help-seeking. Long symptom duration and parent rated impairment was associated with increased risk of reporting several barriers to help-seeking.

**Conclusion:**

Parents seeking help for their child’s mental health encounter numerous barriers that could explain part of the treatment gap and long duration of mental health problems in children prior to referral to CAMHS.

## Introduction

Half of all lifetime mental health disorders have their onset in childhood and adolescence [1]. If untreated, childhood mental health disorders have a high level of persistence [2]. However, studies from several countries have shown that only a minority of children and adolescents with impairing mental health disorders are in contact with specialist child and adolescent mental health services (CAMHS) [3] and that children who do come in contact with CAMHS often have had mental health problems for years before being referred [4, 5]. Severity of symptoms [6], comorbidity [7] and persistency of symptoms [8] have all been identified as predictors of contact with CAMHS. However, Ford et al. found that among children who had persistent symptoms for three years, 61.6% had not accessed CAMHS [8]. Similarly Lempinen et al. found that only half of the children with reported multiple mental health problems were in contact with services [7].

These findings raise questions about why so many children with persistent mental health problems do not access services. In order to narrow the gap between the need for services and access to services it is important to understand not only what determines service access [9] but also what constitutes barriers to accessing services. Help-seeking for children and adolescents is generally led by parents [10, 11] and parental perception of the child’s mental health problems is associated with service use [12–14]. Therefore, it is highly relevant to investigate what parents perceive as barriers to accessing services for their child’s mental health problems in order to identify target areas for minimizing the gap between service needs and service provision [15].

A systematic review by Reardon et al. [15] identified only three quantitative studies on parent perceived barriers to accessing mental health services in a European setting [15], focusing on either a specific ethnic group [16] or specific mental disorders (ADHD and conduct disorder) [17, 18]. In more recent studies on help-seeking for children with anxiety parents have highlighted the need to keep pushing services to ensure professional support for their child’s mental health problems [13, 14, 19].

In order to tailor mental health services to the varying needs of children with different mental health problems, we need to investigate the perceived barriers among parents of children of different ages and across different common mental health disorders [15].

The aim of this study is to investigate parent perceived barriers to accessing services for their child’s mental health problems. In addition, to investigate if parent perceived barriers vary according to the age of the child, type and severity of the child’s mental health problem and duration of the mental health problems.

## Methods

### Design

The study was a cross-sectional study, where parents of all children aged 2–17 years referred to outpatient CAMHS were invited to participate. Information about barriers to help-seeking was collected using the Children’s services interview [20].

### Setting

The study was conducted at the only specialized CAMHS center in the North Denmark Region. The catchment area of the center covers both rural and urban areas with a total population of around 600.000. The center assesses and treats all psychiatric disorders for children aged 0–18 years of age. Denmark has a tax-funded public sector meaning that all public mental health services are free of charge. Access to CAMHS requires a formal referral from either a general practitioner or another medical doctor, an educational psychologist, or a caseworker in social services.

### Eligibility criteria and recruitment

All parents or foster parents (henceforth just referred to as parents) who had a child referred between 1st of July and 31st of December 2018 were invited to participate in the study.

Participants were excluded from the study, if the child was not a Danish resident, contact information on the parents was unknown, or the parents did not understand Danish.

When the CAMHS center received a referral letter for the child, an invitation to participate in the study was sent electronically to the parent along with a consent form.

### Procedures

After accepting to participate in the study, parents completed an electronic questionnaire comprising background information and the extended version of the Strength and difficulties questionnaire (SDQ) [21]. In addition, a research psychologist (CMJ) or medical doctor (ASH) conducted a telephone interview on barriers encountered in accessing services for their child’s mental health problem using the Children’s services interview [20].

Study participants were contacted up to three times to remind them to fill out the electronic questionnaire, and likewise three attempts were made to schedule the telephone interview before the data was accepted as missing. The median time from referral to the telephone interview was 27 days.

### Measures

*The Children’s service interview* is a service use measure, developed to be administered by telephone [20]. It has been shown to be at least moderately valid and reliable in a clinical sample when administered by lay interviewers [20]. The first section uses a semi-structured approach to inquire about interventions received for the child’s mental health problems and the satisfaction with these within a set period. The second section examines reluctance to seek help and perceived barriers to accessing services. If a parent endorses a barrier, they are asked to specify how they experienced the specific barrier [20]. The length of the interview was between 30 and 60 min.

*The extended version of the Strength and difficulties questionnaire (SDQ) *[21] is a short behavioral screening questionnaire about mental health symptoms and their impact [21]. The SDQ is well validated [22], has satisfactory to strong psychometric properties [22–24] and is useful in clinical research [25]. Danish norms for SDQ scores exist [26].

Referral source and referral diagnosis was collected from the referral letters. Previous contacts with the regional CAMHS center and current referral decision were documented from the medical records.

*Referral diagnoses* were divided into (I) Neurodevelopmental disorders (Attention deficit disorders (ADHD/ADD), autism spectrum disorders (ASD), and tic disorders), (II) Emotional disorders (anxiety disorders, affective disorders, and eating disorders) and (III) Others (psychosis, conduct disorders, attachment disorders, personality disorders, and unspecified mental health problems). The referral diagnoses used were the referral reason stated on the referral letter to CAMHS, and therefore not verified CAMHS diagnoses.

### Analyses

Continuous variables are presented with medians and interquartile ranges (IQR), and categorical variables with frequencies and percentages. T-test were conducted for continuous variables and Fischer’s exact test for categorical variables. All statistical tests were two-tailed.

To examine for representativeness, we compared the study sample to all eligible referrals in the inclusion period. For continuous variables, we could not separate the study sample from all eligible referrals, and the values given, therefore also include the study sample.

Logistic regression was used to test for specific barriers’ association with age, referral diagnosis, symptom duration prior to referral, and impairment score above the norm on the SDQ. All logistic regression analyses were tested with bootstrap using 200 repetitions. Adjusted logistic regression analyses were controlled for sex, placement outside the home, previous psychiatric assessment, age, referral diagnosis, symptom duration, and impairment above the norm on SDQ.

The level of statistical significance was set at 5% for all analyses. All statistical analyses were executed using Stata Statistical Software: Release 15. College Station, TX: StataCorp LLC.

To examine reasons for perceived barriers, the specification of why a barrier was endorsed was systematically examined by independent coders (ASH and a graduate level research assistant) using semantic thematic analysis [27]. This qualitative method for analyzing the responses was selected, despite the data not fulfilling the criteria for qualitative data, as it is not verbatim transcriptions, to better understand the different reasons as to why parents perceived different barriers to exist. Any differences in identified themes between the two coders were discussed and a consensus agreement was reach on prominent themes.

## Results

### Participants

**Fig. 1:**
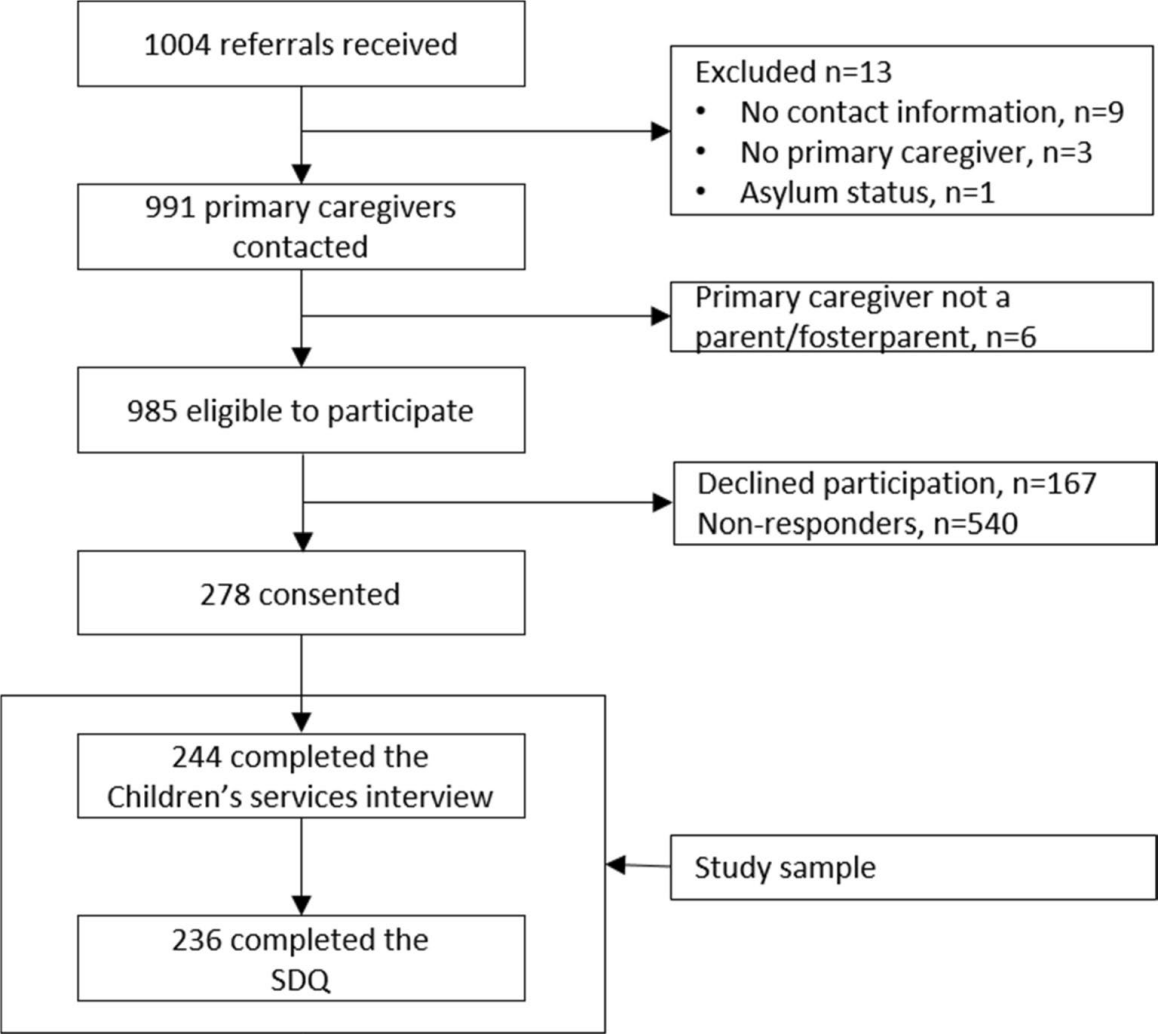
Flowchart for inclusion of participants in the study

Recruitment for the study is illustrated in the flowchart in Fig. 1. 985 primary caregivers were eligible to participate in the study. Of these 278 (28.2%) consented, but only 244 (87.8%) completed the telephone interview. For the 244 completed interviews, 236 also completed the electronic questionnaire.

### Representativeness

When comparing the children of the participating parents with children referred within the study period, but not included in the study there were no statistically significant differences in age, sex distribution, placement outside the home or referral reason between the two groups (Table 1). However statistically significantly fewer were referred by educational psychologist (28.2 vs. 33.6%, p = 0.04).

**Table 1:** Characteristics of the sample

	Study sample (N = 244)	Non-participating referrals (N = 741)	P-value
*Child*			
Age, median (IQR)	12.8 (9.8–14.6)	13.5^a^ (9.8–15.8)	–
Boys, n (%)	148 (60.7)	403 (54.4)	0.09
Lives with both parents, n (%)	138 (56.6)	–	–
Placed outside the home, n (%)	21 (8.6)	55 (7.4)	0.58
Placed in special education program, n (%)	43 (17.6)	–	–
Born in Denmark, n (%)*	229 (97.0)	–	–
≥ 1 parent has ≥ 3 years of higher education, n (%) *	126 (53.4)	–	–
Both parent in regular employment, n (%)*	123 (52.1)	–	–
*Psychiatric history*			–
Parent reported duration of mental health problems in the child, median years (IQR)	5.6 (3.1–9.0)	–	–
Previously assessed for a mental health disorder	87 (35.7)	290 (39.1)	0.36
*Referral*			
Referral diagnosis**, n (%)			
Neurodevelopmental disorders	146 (59.8)	420 (56.7)	0.41
Emotional disorders	74 (30.3)	193 (26.1)	0.21
Other	32 (13.1)	170 (17.2)	0.12
Referral source, n (%)			
General practitioner	122 (50.0)	421 (56.6)	0.06
Educational psychologist	82 (33.6)	197 (28.2)	0.04
Other***	40 (16.4)	128 (17.3)	1.00
*Symptom severity (SDQ)*			
Total SDQ problem score, n (% above the norm)*	205 (86.9)	–	–
SDQ impairment score, n (% above the norm)*	202 (85.6)	–	–

^a^For age, it was not possible to separate the study sample from non-participating referrals. Median age indicated is for all eligible referrals in the study period (N = 985)

*236 completed the questionnaire with background information and the SDQ

**Total N > 244 due to co-morbidity. 8 with both neurodevelopmental and emotional disorder as referral reason

***Other: Pediatrician, social services or other mental health services

### Characteristics of the referred children in the sample

As seen by Table 1 the median age of the referred children was 12.8 years of age (IQR 9.8–14.6) and 60.7% were boys. The majority were born in Denmark (97.0%) and 8.6% were placed outside of the home. As for schooling 17.6% were placed in special education programs.

The median duration of parent reported child mental health problems prior to referral to CAMHS was 5.6 years (IQR 3.1–9.0), and just under one third (29.9%) had previously been assessed for a mental health disorder. The majority of children were referred for neurodevelopmental disorders (59.8%), 30.3% for emotional disorders and 13.1% for other disorders. Most of the referred children had a total SDQ problem score (86.9%) and an SDQ impairment score (85.6%) above the norm.

### Parents’ general attitudes towards help and services received prior to referral to CAMHS

“Hesitation to seek help from professionals” was reported by 41.0% (n = 100) of the parents. Thematic analysis of the specifications for hesitation revealed difficulties in differentiating normal development from mental health problems and needing time to accept that the problem is beyond what you can handle yourself as prominent themes. Some parents also stated that needing help to handle their child’s mental health problems made them feel like they failed as a parent.

The majority (62.7%, n = 153) of parents reported that child mental health interventions before referral to CAMHS have had some impact. Being listened to and taken seriously by a professional made a difference as did speedy referral to relevant services and receiving services directed towards both the child and the family.

### Parent reported barriers

**Fig. 2:**
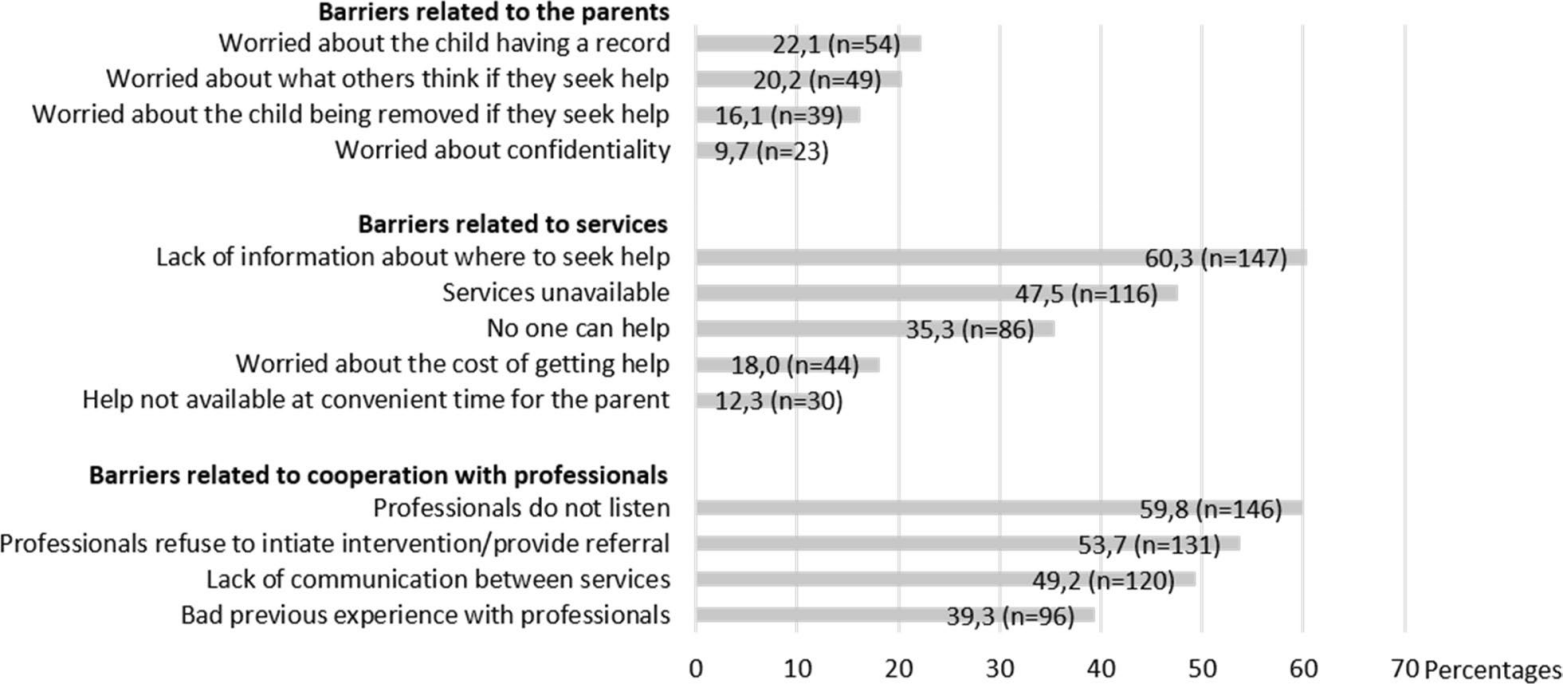
Percentage of parents reporting barriers to help-seeking for their child’s mental health problems

Figure 2 shows the percentage of parents who reported encountering different barriers throughout their help-seeking pathway.

#### Barriers related to the parents

The most frequent barriers related to the parents were worries about the child having a record (n = 54, 22.1%) and worries about what other people would think if they sought help (n = 49, 20.2%). Concerns about what it would mean for the child to have a record were related to worries about their child being labelled and subsequent stigmatization and consequences for future employment and insurance.

Worries about what others might think if they sought help for their child were due to negative attitudes of relatives and friends towards child mental health disorders and mental health services and concerns that others would judge their parenting skills and, in some cases, also concern from the child about negative attitudes from their peers.

Worry about the child being removed from the home (n = 39, 16.1%) was primarily a barrier to seeking help from social services prior to referral. Parents expressed concerns about being viewed as a bad parent and being blamed for the child’s mental health problems when seeking help.

#### Barriers related to services

The most frequent barrier overall was lack of information about where to seek help (n = 147, 60.3%). Parents did not know what services to contact to access help, they felt that services often “passed the ball around” and they missed information about their rights regarding help and support for their child. Many parents stated that it required many resources to be proactive in help-seeking.

Just below half of the parents had experienced that relevant services were not available to them (n = 116, 47.5%). This was due to long waiting times, lack of flexibility from services, rejection of referrals to CAMHS, and the experience that it was hard to access services in other sectors without an assessment from CAMHS.

About one third (n = 86, 35.3%) of parents perceived it as a barrier that no one in the system could help their child. Lack of knowledge from the professionals involved and a perception of lack of resources within services was specified. Some parents also had the feeling that it was difficult to obtain the right help if their child did not display prototypical symptoms of a mental health disorder or the child’s symptoms were either too mild or too severe to access specific services.

Worries about the cost of getting help (n = 44, 18.0%) and help not being available at a convenient time, was only reported by a minority of the parents and were both linked with parents having to take time off from work to attend appointments.

#### Barriers related to cooperation with professionals

The most frequently endorsed barrier related to cooperation with professionals was the perception that professionals did not listen (n = 146, 59.8%). This was across all sectors and across different professions. Parents often felt that their observations were less valuable than observations by professionals, and many parents felt blamed by professional for the child’s difficulties.

More than half of parents (n = 131, 53.7%) experienced that professionals refused to initiate an intervention or provide a referral to other services. This included teachers not wanting to refer to educational psychologists, disagreement between professionals about who should refer the child to CAMHS, professionals opposing referral to CAMHS and CAMHS rejecting referrals.

Lack of communication within or between services was also a barrier for half the parents (n = 129, 49.2%). They experienced information getting lost in transition between services, and many parents experienced a large overturn of professionals involved in their child’s case with insufficient handover. GPs did not have access to information from educational and social services and parents felt they had to be the carrier of information between different services.

Bad previous experiences with a professional was reported as a barrier by 39.3% (n = 96). Lack of follow through from professionals on agreed upon interventions and the perception of being blamed for their child’s mental health problems was specified for this barrier. In addition, previous experiences of professionals downplaying the child’s mental problem made parents hesitant to pursue help again.

### Factors influencing parent perceived barriers

#### Barriers’ association with age

No barriers were associated with the age of the referred child in the adjusted regression model (Table 2).

**Table 2:** Association between parent reported barriers, age and referral diagnosis

	Age^a^				Referral diagnosis^b^	
	10–14 years		15–17 years		Emotional disorders	
	Crude	Adjusted	Crude	Adjusted	Crude	Adjusted
*Barriers related to the parents*						
Worried about the child having a record	1.29 (0.59–2.82)	1.46 (0.58–3.71)	0.95 (0.37–2.45)	1.05 (0.35–3.14)	0.93 (0.40–2.16)	1.02 (0.36–2.89)
Worried about what others think if they seek help	0.57 (0.29–1.14)	0.68 (0.32–1.46)	*0.32* (*0.10*–*0.97*)	0.37 (0.11–1.25)	*0.37* (*0.14*–*0.98*)	*0.21* (*0.05*–*0.88*)
Worried about the child being removed if they seek help	0.65 (0.29–1.43)	0.62 (0.25–1.51)	0.48 (0.15–1.46)	0.40 (0.12–1.37)	0.53 (0.18–1.54)	0.70 (0.21–2.35)
Worried about confidentiality	0.68 (0.22–2.06)	0.71 (0.22–2.29)	0.59 (0.15–2.39)	0.43 (0.11–1.66)	0.79 (0.25–2.47)	1.35 (0.28–6.47)
*Barriers related to services*						
Lack of information about where to seek help	*2.16* (*1.26*–*3.71*)	2.10 (0.99–4.47)	1.30 (0.63–2.68)	1.32 (0.54–3.23)	0.73 (0.39–1.35)	0.69 (0.25–1.86)
Services unavailable	1.43 (0.72–2.85)	1.29 (0.65–2.57)	0.71 (0.32–1.61)	0.71 (0.30–1.70)	1.19 (0.65–2.18)	2.06 (0.84–5.05)
No one can help	1.41 (0.74–2.70)	1.34 (0.66–2.74)	1.09 (0.49–2.46)	1.13 (0.43–2.94)	0.76 (0.38–1.50)	0.62 (0.25–1.58)
Worried about the cost of getting help	0.72 (0.34–1.51)	0.60 (0.24–1.48)	0.76 (0.26–2.21)	0.65 (0.22–1.94)	1.28 (0.60–2.72)	1.36 (0.52–3.57)
Help not available at convenient time	1.37 (0.45–4.19)	1.17 (0.36–3.77)	0.88 (0.21–3.64)	0.90 (0.21–3.96)	0.74 (0.27–2.02)	1.43 (0.32–6.40)
*Barriers related to cooperation with professionals*						
Professionals do not listen	0.96 (0.51–1.82)	0.86 (0.39–1.92)	0.79 (0.37–1.70)	0.77 (0.33–1.79)	0.70 (0.39–1.29)	0.81 (0.31–2.08)
Professionals refuse to initiate intervention/provide referral	1.45 (0.75–2.80)	1.61 (0.82–3.17)	0.52 (0.24–1.11)	0.64 (0.29–1.42)	0.60 (0.33–1.09)	1.00 (0.39–2.57)
Lack of communication between services	1.30 (0.71–2.39)	1.18 (0.62–2.26)	0.93 (0.42–2.05)	0.96 (0.39–2.36)	0.54 (0.29–1.01)	0.76 (0.30–1.92)
Bad previous experience with professionals	0.94 (0.52–1.73)	0.85 (0.40–1.80)	0.74 (0.36–1.54)	0.75 (0.33–1.74)	0.60 (0.30–1.22)	1.10 (0.46–2.59)

^a^Reference: Age < 10 years; Adjusted for sex, placement outside the home, symptom duration, impairment score on SDQ and referral diagnosis

^b^Reference: Neurodevelopmental disorder; Adjusted for sex, age, placement outside the home, symptom duration and impairment score on SDQ.

#### Barriers’ association with referral diagnosis

The only barrier statistically significantly associated with referral diagnosis was “Worried about what others think if they seek help”. Parents of children referred for emotional disorders were less likely to report this barrier compared to parents of children referred for neurodevelopmental disorders [Adj. OR 0.21 (95%CI 0.05–0.88)] (Table 2).

#### Barriers’ association with symptom duration prior to referral

As seen in Table 3, parents of children with symptoms for > 5 years endorsed the barrier “Worried about the child being removed if they seek help” more frequently compared to parents of children referred within the first year of symptoms [Adj. OR 3.32 (95%CI 1.03–10.76)].

**Table 3:** Association between parent reported barriers, symptom duration and parent reported impairment

	Symptom duration prior to referral^a^				Impairment on SDQ^b^	
	1–5 years		> 5 years		Above the norm	
	Crude	Adjusted	Crude	Adjusted	Crude	Adjusted
*Barriers related to the parents*						
Worried about the child having a record	0.63 (0.19–2.02)	0.58 (0.14–2.37)	0.99 (0.34–2.88)	0.96 (0.26–3.49)	2.40 (0.77–7.47)	2.30 (0.70–7.59)
Worried about what others think if they seek help	0.99 (0.30–3.28)	0.70 (0.16–3.08)	0.93 (0.29–2.96)	0.91 (0.23–3.60)	*4.75 (1.50–15.06)*	*5.76 (1.79–18.48)*
Worried about the child being removed if they seek help	*4.79 (1.65–13.90)*	3.06 (0.91–10.28)	*4.85 (1.77–13.34)*	*3.32 (1.03–10.76)*	3.26 (0.88–12.10)	3.08 (0.75–12.59)
Worried about confidentiality	–	–	–	–	*3.83 (1.44–10.15)*	*3.57 (1.15–11.10)*
*Barriers related to services*						
Lack of information about where to seek help	*3.44 (1.14–10.38)*	*3.35 (1.03–10.93)*	*4.91 (1.64–14.69)*	*4.62 (1.46–14.62)*	1.41 (0.62–3.17)	1.23 (0.55–2.74)
Services unavailable	1.14 (0.47–2.77)	0.72 (0.25–2.08)	1.42 (0.58–3.47)	1.09 (0.37–3.19)	1.83 (0.81–4.17)	1.81 (0.74–4.42)
No one can help	1.50 (0.55–4.09)	1.19 (0.38–3.70)	1.87 (0.70–4.99)	1.65 (0.49–5.52)	*2.87 (1.08–7.65)*	*2.91 (1.00–8.42)*
Worried about the cost of getting help	3.07 (0.91–10.35)	2.79 (0.64–12.11)	2.30 (0.68–7.78)	2.26 (0.57–9.83)	1.26 (0.38–4.21)	1.19 (0.38–3.72)
Help not available at convenient time	–	–	–	–	0.71 (0.20–2.49)	0.50 (1.44–1.75)
*Barriers related to cooperation with professionals*						
Professionals do not listen	*3.97 (1.37–11.49)*	2.91 (0.90–9.41)	*4.30 (1.44–12.80)*	2.85 (0.99–8.22)	*2.79 (1.29–6.06)*	*2.56 (1.14–5.72)*
Professionals refuse to initiate intervention/provide referral	1.56 (0.61–3.96)	1.14 (0.34–3.74)	2.52 (0.99–6.37)	1.86 (0.57–6.11)	*2.33 (1.03–5.26)*	2.16 (0.92–5.06)
Lack of communication between services	3.07 (0.96–9.85)	2.01 (0.61–6.60)	*3.33 (1.12–9.91)*	1.83 (0.56–5.98)	*2.65 (1.16–6.06)*	*2.50 (1.02–6.14)*
Bad previous experience with professionals	*3.88 (1.07–13.98)*	2.83 (0.70–11.48)	*6.11 (1.77–21.05)*	*4.78 (1.17–19.45)*	1.90 (0.82–4.39)	1.62 (0.67–3.93)

^a^Reference: Symptom duration < 1 year;

Adjusted for sex, age, placement outside the home, previous psychiatric assessment, impairment score on SDQ and referral diagnosis.

^b^Reference: SDQ impairment score within the norm (80th percentile);

Adjusted for sex, age, placement outside the home, previous psychiatric assessment, symptom duration and referral diagnosis.

–, not possible to calculate OR for “Worried about confidentiality” and “Help not available at convenient time” according to symptom duration.

Both parents of children with symptoms for 1–5 years [Adj. OR 3.35 (1.03–10.93)] and > 5 years prior to referral [Adj. OR 4.62 (1.46–14.62)] had increased reporting of the barrier “Lack of information about where to seek help” compared to parents of children referred within the first year, and this finding was most pronounced for the parents of children with symptoms for > 5 years.

The barrier “Bad previous experience with professionals” was only statistically significantly associated with symptom duration for parents of children with symptoms for > 5 years [Adj. OR 4.78 (95%CI 1.17–19.45)].

#### Barriers association with SDQ reported impairment

Parents of children who scored above the norm for impairment on the SDQ were more than 5 times as likely to report “Worried about what others think if they seek help” as a barrier [Adj. OR 5.76 (95%CI 1.79–18.48)] and were also more likely to report “Worried about confidentiality” as a barrier [Adj. OR 3.57 (95%CI 1.15–11.10)] compared to parents of children who scored within the norm (Table 3). Also, they were more likely to report the perception that “No one can help” [Adj OR 2.91 (95%CI 1.00–8.42)].

For barriers related to cooperation with professionals, there was an association with impairment for “Professionals do not listen” [Adj. OR 2.56 (95%CI 1.14–5.72)] and “Lack of communication between services” [Adj. OR 2.50 (95%CI 1.02–6.14)] which were both more frequently endorsed by parents of children scoring above the norm score for impairment.

## Discussion

The aim of this study was to investigate parent perceived barriers to accessing services for their child’s mental health problems in order to identify potential areas for improvement of access to CAMHS.

In this study longer symptom duration was found to increase the risk of reporting several barriers. Parent rated severity, as measured by impairment on the SDQ, was associated with the highest number of barriers. Due to the cross-sectional design of the study and only having parent ratings for impairment on the SDQ, it is not possible to conclude if this is due to parents of children with more severe problems encountering more barriers, or if parents who encounter many barriers in their help-seeking rate their child’s problems as more impairing. Studies investigating the effect of parent’s experiences of being included in decision making regarding their child’s mental health treatment have shown an association with higher level of parent-reported improvement of the child’s mental health problem [28, 29]. It is therefore likely that the perception of negative attitudes from others and the perception that professionals are dismissive in the help-seeking process can lead to parents experiencing the child’s mental health problems as more impairing.

In the specifications for endorsing different barriers, three over-all themes arose: (1) Lack of knowledge about child mental health and help-seeking pathways, (2) Stigmatization and parent blame (3) Challenges of multi-agency collaboration.

### Lack of knowledge about child mental health and help-seeking pathways

Lack of information about where to seek help was reported by the majority of parents. In a systematic review from 2017 Reardon et al. found this barrier to be reported by 14–75% of parents in quantitative studies which was corroborated by a number of qualitative studies [15]. We found an association between parents reporting lack of information on where to seek help as a barrier and symptom duration prior to referral. It might be that children who have long delays in time-to-referral to CAMHS have displayed more mixed symptomology, making it harder to recognize their symptoms as a mental health problem and thus making it difficult to direct the parents to the correct services. This might also explain the higher risk for reporting this barrier for parents of children aged 10–14 compared to parents of children referred before the age of 10. Mental health problems of children referred between the ages of 10–14 might have been less clear in early childhood, making it harder for both the parents and professionals to find relevant services. Another explanation could be a lack of knowledge about child mental problems and child mental health services among professionals in primary settings. The parent’s first help-seeking contact, when a child displays mental health problems is most often general practitioners or teachers [17, 30–32], but they often lack training in child mental health [33, 34] and might not have sufficient knowledge of which services are available or when it would be appropriate to refer to specialized CAMHS [33]. It is worrisome that even among parents, who obtain a referral to CAMHS, so many found lack of information about where to seek help a barrier. Difficulties in obtaining information about where to seek help might deter less resourceful parents from seeking out timely and relevant help for their child’s mental health problems.

### Stigmatization

Mental health disorders are still stigmatized [35] and this also applies to child mental health disorders [36], where parents of the afflicted child also experience stigmatization by association [37, 38].

In previous studies on parent perceived barriers, the most commonly reported barrier related to concerns about consequences of help seeking has been the perceived negative attitudes among other people [15].

Worrying about the child having a record and worrying about what other people would say if they sought help was reported as a barrier to help-seeking by a fifth of the participating parents in this study. This was due to concerns about both stigmatization of the child and judgement of parenting skills. Studies have shown a higher level of parental associated stigma for child mental health disorders [39] and a higher attribution of parental blame by the public for child mental health disorders [40] compared to physical disorders. This is true for both emotional and neurodevelopmental disorders, but most pronounced for neurodevelopmental disorders [40]. These findings are in line with this study, were parents of children referred for emotional disorders were less likely to endorse the barrier “Worry about what others think if they seek help” compared to parents of children referred for neurodevelopmental disorders. This might be due to children referred for neurodevelopmental disorders displaying more externalizing symptoms, which are more likely attributed to parental failure [40]. Also there is a general public opinion that neurodevelopmental disorders are being overdiagnosed more so than emotional disorders [41]. Perceived attribution of parental blame was not only reported from the public but also as an issue in the cooperation with professionals. This has previously been reported by Johnson and colleagues [42]. In their study among social workers, psychologists, and child psychiatrists one in five agreed that a child’s mental health problems could be attributed to deficient parenting and half showed some agreement with attribution of parental responsibility [42]. Professionals who were trained in child psychiatry and neuropsychology were less likely to agree with parental attribution of responsibility [42]. Also, professionals who assigned responsibility for child mental health problems to deficient parenting were less likely to refer the child to services [42], which was a barrier reported by more than half the parents in our study. It is therefore possible that professionals’ reluctance to initiate interventions or refer the child to other services could in part be due to the attribution of the child’s mental health problems to parenting problems. This could result in too large a focus on interventions aimed at parenting skills as supposed to targeting the child’s mental health problem.

### Challenges of multi-agency collaboration

There has been increasing focus in child mental health policy on multi-agency collaboration across healthcare, educational, and social service sectors, with the aim of providing early interventions for children with mental health problems [17, 43, 44]. However, several barriers to multi-agency collaboration have been identified [45] and this study supports that the multi-agency nature of child mental health services creates barriers for parents in their help-seeking. Lack of information about where to seek help might in part be due to difficulties navigating between services spread across different sectors for both parents and the professionals. In addition, legislation within educational services, healthcare services and social services are not always aligned making coordination and collaboration within child mental health care more challenging. For a multi-agency collaboration to function well, it is important that the involved services possess enough resources and competencies to provide relevant interventions within their field. Unavailability of services is a frequently reported structural barrier in studies on parent perceived barriers [15] and inadequate resourcing is the most commonly cited barrier to multi-agency collaboration [45]. In this study almost half of the participants reported unavailability of services as a barrier. In addition, more than half reported professionals refusing to initiate interventions or provide a referral. This could be a result of both lack of knowledge and lack of resources. Lack of communication between services was another frequently reported barrier in this study. This is another potential barrier to successful multi-agency collaboration [45–47]. Cooper et al. advocate for the need for adequate channels of communication between different services and a named link person to improve the collaboration and communication between services [45]. This could tackle some of the challenges raised in our study of the parent becoming the carrier of information between different professionals, which is an additional demand on parents already strained by their child’s mental health problems.

## Strengths and limitations

To our knowledge this study is the largest study performed among service users of CAMHS and the only study investigating a population that is representative of the population referred to outpatient CAMHS. This enabled us to investigate differences in barriers associated with age, type of problem, severity and chronicity. In addition, the supplementation of quantitative data with more qualitative specifications of why the participants endorsed a specific barrier is a strength.

However, several limitations to this study should be mentioned. Firstly, the cross-sectional design does not allow for drawing conclusions about causality for the perceived barriers. In addition, only a quarter of the eligible participants were included in the study, which leads to a risk of selection bias. However, we did compare the study sample to all referrals in the inclusion period and the only statistically significant difference was that less children had been referred by educational psychologists.

Another limitation was that participants were included in the study prior to formal assessment by CAMHS, wherefore the diagnoses used to differentiate between neurodevelopmental disorders and emotional disorders were not verified CAMHS diagnoses and there is only a modest correlation between referral diagnosis and final CAMHS verified diagnoses[48].

In addition, the telephone interviews, were not recorded and transcribed verbatim which is a weakness regarding the qualitative data. However, it was a strength to this study that the interviewers had clinical experience compared to using lay interviewers with regards to ensuring that the collected information correlated to help-seeking for mental health problems.

This study only included parents as informants, and it would be relevant to also explore the views of children/adolescents as well as the barriers perceived by professionals to get a more complete picture of barriers to efficient help-seeking.

Importantly, as this study focused only on parents of children referred to specialized CAMHS, it is not possible to deduce anything about which parent-perceived barriers might deter some parents from obtaining a referral to CAMHS, despite the child having a treatment need or barriers encountered by parents of children with milder mental health problems not prompting referral to specialized CAMHS.

## Conclusion and clinical implications

Parents of children with mental health problems still encounter numerous barriers when seeking help. Despite increasing knowledge of mental disorders, stigma and parental attribution of responsibility are still important barriers. Parents experience of professional as dismissive is also deterring to their help-seeking efforts. Targeted training in child mental disorders to professionals working with children and adolescents might help to address these issues. There is a need to increase the knowledge of how child mental health services are organized both in the public and among professionals. In order to provide early and comprehensive services to children with mental health problems it is important to have sufficient resources across healthcare, education, and social services with focus on developing appropriate channels for information sharing across service sectors. Increasing the availability of consulting child mental health specialists in primary healthcare, educational- and social service setting could help to increase the knowledge base of professionals in these settings and facilitate better multi-agency collaboration.

Future research should focus on evaluating the effects of interventions aimed at diminishing the identified barriers and how the involved children, parents and professionals perceive these interventions.

## Data Availability

The datasets for the current study are available from the corresponding author on reasonable request.

## Abbreviations

CAMHS: Child and adolescent mental health services
SDQ: The Strength and Difficulties Questionnaire
ADHD: Attention deficit hyperactivity disorder
ASD: Autism spectrum disorder
